# Analysis of multiple chronic disease characteristics in middle-aged and elderly South Koreans by exercise habits based on association rules mining algorithm

**DOI:** 10.1186/s12889-023-16099-4

**Published:** 2023-06-26

**Authors:** Yingcheng Huang, Yaqi Su, Yonghyun Byun, Youngil Lee, Sangho Kim

**Affiliations:** 1 Korea University, Sejong, South Korea; 2 Dankook University, Cheonan, South Korea; 3 University of West Florida, Pensacola, FL USA

**Keywords:** Multiple chronic disease, Association rules mining algorithm, Exercise

## Abstract

**Background:**

The term, “multiple chronic diseases” (MCD), describes a patient with two or more chronic conditions simultaneously at the same time. Compared with general chronic diseases, it is linked to poorer health outcomes, more difficult clinical management, and higher medical expenses. Several existing MCD guidelines support a healthy lifestyle including regular physical activities but do not include specific exercise therapy recommendations. This study aimed to understand the prevalence and model of MCD in middle-aged and elderly South Koreans by comparing MCD characteristics with exercise habits, to provide a theoretical basis for the implementation of exercise therapy in these patients.

**Methods:**

The data of 8477 participants aged > 45 years from the “2020 Korean Health Panel Survey” were used to analyze the current status of MCD in the middle-aged and elderly. The Chi-square test for categorical variables and the t-test for continuous variables. the used software was IBM SPSS Statistics 26.0 and IBM SPSS Modeler 18.0.

**Results:**

In this study, the morbidity rate of MCD was 39.1%. Those with MCD were more likely to be female (p < 0.001), seniors over 65 years of age (p < 0.001), with low education level, no regular exercise behavior (p < 0.01). Chronic renal failure (93.9%), depression (90.4%), and cerebrovascular disease (89.6%) were the top three diseases identified in patients with MCD. A total of 37 association rules were identified for the group of individuals who did not engage in regular exercise. This equated to 61% more than that of the regular exercise group, who showed only 23 association rules. In the extra association rules, cardiovascular diseases (150%), spondylosis (143%), and diabetes (125%) are the three chronic diseases with the highest frequency increase.

**Conclusions:**

Association rule analysis is effective in studying the relationship between various chronic diseases in patients with MCD. It also effectively helps with the identification of chronic diseases that are more sensitive to regular exercise behaviors. The findings from this study may be used to formulate more appropriate and scientific exercise therapy for patients with MCD.

## Background

The occurrence and prevalence of chronic diseases are a serious threat to human life and health. These have always been the most important causes of death. A total of 41 million people die from chronic diseases annually. This is equivalent to 71% of all deaths worldwide [1]. When two or more chronic diseases coexist in the same patient, it increases the difficulty of diagnosis, evaluation, and treatment, increases the risks of adverse health outcomes, consumes more medical resources, and reduces the patient’s quality of life [2]. The term “multiple chronic diseases” (MCD) describes such patients, regardless of the types of chronic disease [3]. Some scholars have proposed that “comorbidity” needs to be clarified in the causal relationship between multiple chronic diseases [4]. In this study, causality is not considered. Instead, MCD is used to describe eligible patients. Our concept is similar to that of “multimorbidity” defined by The Word Health Organization (WHO) in 2008 [5]. Previous studies have demonstrated that individuals with MCD not only frequently face poorer health outcomes [6, 7] but also have greater rates of hospital readmission [8] and significantly higher healthcare costs [9]. In the United States, nearly 80% of Medicare spending is devoted to patients with four or more chronic conditions, with costs increasing exponentially as the number of chronic conditions increases [10].

In recent years, as the COVID-19 epidemic has affected normal lifestyles and life rhythms to varying degrees, some patients with chronic diseases have been affected by unhealthy lifestyles, such as reduced exercise and increased sugar intake, which further increases their risks of disease progression [11].

In May 2020, WHO assessed the global status of medical resources and services for chronic diseases. According to the WHO survey findings, services for the treatment of hypertension were partially or completely interrupted during COVID-19 in more than half (53%) of the nations studied, 49% of countries discontinued treatment services for diabetes and related complications, 31% of nations stopped providing emergency cardiovascular services, and 42% of countries stopped providing cancer treatment services [12]. All the evidence points to patients with chronic diseases, especially those with MCD, having had increased risks and treatment expenses during the COVID-19 pandemic. Hence, in addition to conventional medical procedures, patients have sought other forms of useful and efficient “medicine.“

“Exercise is medicine” was initially advocated by Hippocrates (460–370 BCE), the father of scientific medicine. Exercise therapy is recommended as the cornerstone of treatment by clinical standards [13] and has been recognized and supported on a global scale by several researchers [14, 15]. An inverse correlation between exercise and the burden of chronic disease is recognized. Those who report higher levels of exercise tend to have lower risks of chronic disease [16]. Exercise is an instant and “risk-free” strategy for decreasing health risks and can be used to treat [17] and prevent [18] a wide variety of chronic conditions. Exercise does not cost anything, and it is available to both men and women of any age and socioeconomic status [19]. Among the research that examined the relationship between physical activities and diseases, few have paid attention to individuals with MCD [20]. We believe that this issue warrants our attention. As chronic diseases mainly occur in middle-aged and elderly individuals, the prevalence of MCD also increases with age [21]. In addition, middle-aged and elderly individuals often encounter various external and internal barriers that hinder their engagement in physical activity and exercise [22]. Therefore, this study targeted middle-aged and elderly South Koreans.

This study aimed understand the association between various chronic diseases by analyzing the basic characteristics of middle-aged and elderly patients with MCD in Korea and compare the differences between MCD characteristics and different exercise habits. The results of this study may contribute to effectively reducing the prevalence and cost of treatment and prevention of MCD.

## Methods

### Research subjects

This study was based on data from the “Korean Health Panel Survey” (KHPS) [23]. The KHPS is a government-approved statistical survey that has been distributed annually since 2008 to generate national-scale data on healthcare use, medical costs, health levels, and health behaviors. Through rigorous screening of 14 741 participants, 8477 participants, after referring to previous studies on age grouping, [24] aged > 45 years, with a complete set of exercise-related data and chronic disease data were included in this study. The data that support the findings of this study are available from the Korean Health Panel Survey. Restrictions apply to the availability of these data which were used under license for the current study. The data is not publicly available. However, data may be available from the corresponding author upon reasonable request and with permission from the Korean Health Panel Survey. The use of data in this study was approved through IRB review by Bioethics Committee of Korea Institute For Health and Social Affairs (KIHASA) (Assignment No. 22-017-00).

### Research variables

Demographics, such as sex, age, and educational level, were obtained from the KHPS for data analysis. Exercise-related daily habits including regular exercise habits, sports-related expenses, sedentary time, and weight management behaviors were considered. Regarding regular exercise, participants responded to the question, “In the past year, have you done any sports or exercise regularly (including walking)?” Those who answered that they were “doing sports or exercise regularly” were regarded as having regular exercise habits. A total of 30 chronic diseases in the KHPS survey were grouped into 14 types based on different characteristics: hypertension, diabetes, chronic liver diseases (including chronic hepatitis, alcoholic hepatitis, and hepatic cirrhosis), gonarthrosis, arthritis (including degenerative arthritis and rheumy arthritis), spondylosis (including disc disorder and other spongy allodynia), cancer (including gastric cancer, colorectal cancer, lung cancer, breast cancer, cervical cancer, thyroid cancer, and other cancers), cardiovascular diseases (angina and myocardial infarction), cerebrovascular diseases (cerebral hemorrhage and cerebral infarction), chronic respiratory diseases (including asthma, pulmonary emphysema, chronic obstructive pulmonary disease, and bronchiectasis), thyroid diseases (including hypothyroidism and hyperthyroidism), depression, Alzheimer’s disease, and chronic renal failure. The height and weight of each participant were used to calculate their body mass index (BMI), which was then used to determine whether they were obese. BMI was calculated as weight(kg)/height^2^ (m), Considering that the occurrence of related morbidities, such as stroke, ischemic heart disease, and diabetes, is three to four times higher in adults classified as obese (BMI > 28) compared to the general population [25], we defined obesity as a BMI > 28 in our study. This study screened for 15 chronic diseases, including obesity.

### Analysis method

Statistical analyses were performed using IBM SPSS Statistics, version 26.0 and IBM SPSS Modeler, version 18.0. Statistical significance was set at a two-tailed p-value of < 0.05. To compare the MCD characteristics of the participants, the chi-square test for categorical variables and t-test for continuous variables were used. The study subjects were divided into two groups, AG (active group: group of middle-aged and older individuals who have regular exercise habits) and IG (inactive group: group of middle-aged and older individuals who do not have regular exercise habits), based on whether they had regular exercise habits, and data mining was conducted separately for both groups.

### Association rules mining algorithm

Data mining refers to the discovery of hidden information and interesting patterns in databases. The Association rules mining algorithm is one of the most important branches of data mining and identifies essential associations and frequent patterns among a set of items in large databases [26]. It derives a set of items with strong rules by utilizing various evaluation scales, and the relationship between these items is expressed as A→ B [27]. Support, confidence, and lift are the three most important evaluation scales that explain the relationship between A and B.

Support refers to the probability that the item sets included in A and B occur simultaneously. The higher the support, the higher the probability of A and B appearing simultaneously in the association rule. The calculation formula is as follows: Support (A→ B) = P (A, B). In this study, we defined “support” as the “probability of patients having A chronic diseases and B chronic diseases among all subjects.”

Confidence refers to the conditional probability of the occurrence of B, under the premise that A appears. Confidence measures the reliability of association rules. If the confidence is high, B is more likely to appear after A, and the association rule is more credible. The calculation formula is as follows: Confidence(A→ B) = P (A,B)/P(A). In this study “confidence” refers to the “probability of having B chronic diseases among patients with A chronic diseases.”

Lift refers to the ratio of the probability of the occurrence of B in the presence of A to the probability of the occurrence of B under any condition, which explains the degree of influence of A on B. The calculation formula is as follows: Lift(A→ B) = P (A,B)/P(A)P(B) or lift(A→ B) = confidence(A→ B)/P(B). In this study, “lift” is defined as the “confidence value divided by the probability of having B chronic diseases.” Given that different chronic diseases have widely differing basal prevalence rates, lift was the most important evaluation scale value in this study.

Apriori is an algorithm for frequent item set mining and association rule learning in relational databases. It explores candidates in two steps: (1) Generate all frequent items. An itemset is frequent when its occurrence exceeds the minimum support, a pre-given threshold; (2) Suppose all frequent “K” have been explored, create “K + 1” based on “K” and keep just frequent “K + 1.”

The first application of association rules mining is buying performance analysis in a supermarket using the Apriori algorithm developed by Agrawal and Srikan in 1994 [28]. Since then, association rule analysis has not only played an important role in commercial data analysis, but has also been successful in finding interesting patterns and associations in many other fields, especially in the past few years, and has been widely used in medical data analysis [29, 30].

## Results

### Participant characteristics

A total of 8477 participants (3272 men and 4750 women), with a mean age of 65.2 ± 10.6 years participated in this study. Of these, 3317 participants (1268 men and 2049 women)had MCD. Table 1 shows the participant characteristics according to MCD.

Significant differences among the characteristics were detected according to MCD (Table 1). Women had a higher prevalence of MCD than men (p < 0.001). There were significant differences in the prevalence of MCD in different age groups (p < 0.001), with the highest prevalence among those over 75 years old (65.4%), followed by 65–75 years old (53.7%), 55–65 years old (26.8%), and 45–55 years old (12.7%). Those with elementary school or below education level were more likely to have MCD (p < 0.001). Those who had no regular exercise habits (p < 0.01) were also more likely to have MCD.

**Table 1:** Participant characteristics

Variables		N(%)^a^	MCD(%)^b^	*P*
**Gender**				0.000
	Male	3727(44.0)	1268(34.0)	
	Female	4750(56.0)	2049(43.1)	
**Age**				0.000
	46–55	1839(21.7)	234(12.7)	
	56–65	2515(29.7)	675(26.8)	
	66–75	2474(29.2)	1329(53.7)	
	> 75	1649(19.5)	1079(65.4)	
	Mean(SD)^c^	65.2(10.6)	70.5(9.0)	0.000
**Education level**				0.000
	Elementary school or below	2517(29.7)	1577(62.7)	
	Middle school	1549(18.3)	700(45.2)	
	High school	2778(32.8)	754(27.0)	
	University or above	1623(19.1)	286(17.6)	
**Regular exercise habits**				0.001
	Yes	4716(55.6)	1774(37.6)	
	No	3761(44.4)	1543(41.0)	
**Total**		8477(100)	3317(39.1)	

a: Data outside the brackets are the number of cases, and the data in brackets are the composition ratio (%)

b: Data outside the brackets are the number of cases, and the data in brackets are percentages (%)

c Data outside the brackets are the means, and the data in brackets are the standard deviation (SD)

### Prevalence of chronic diseases

**Fig. 1:**
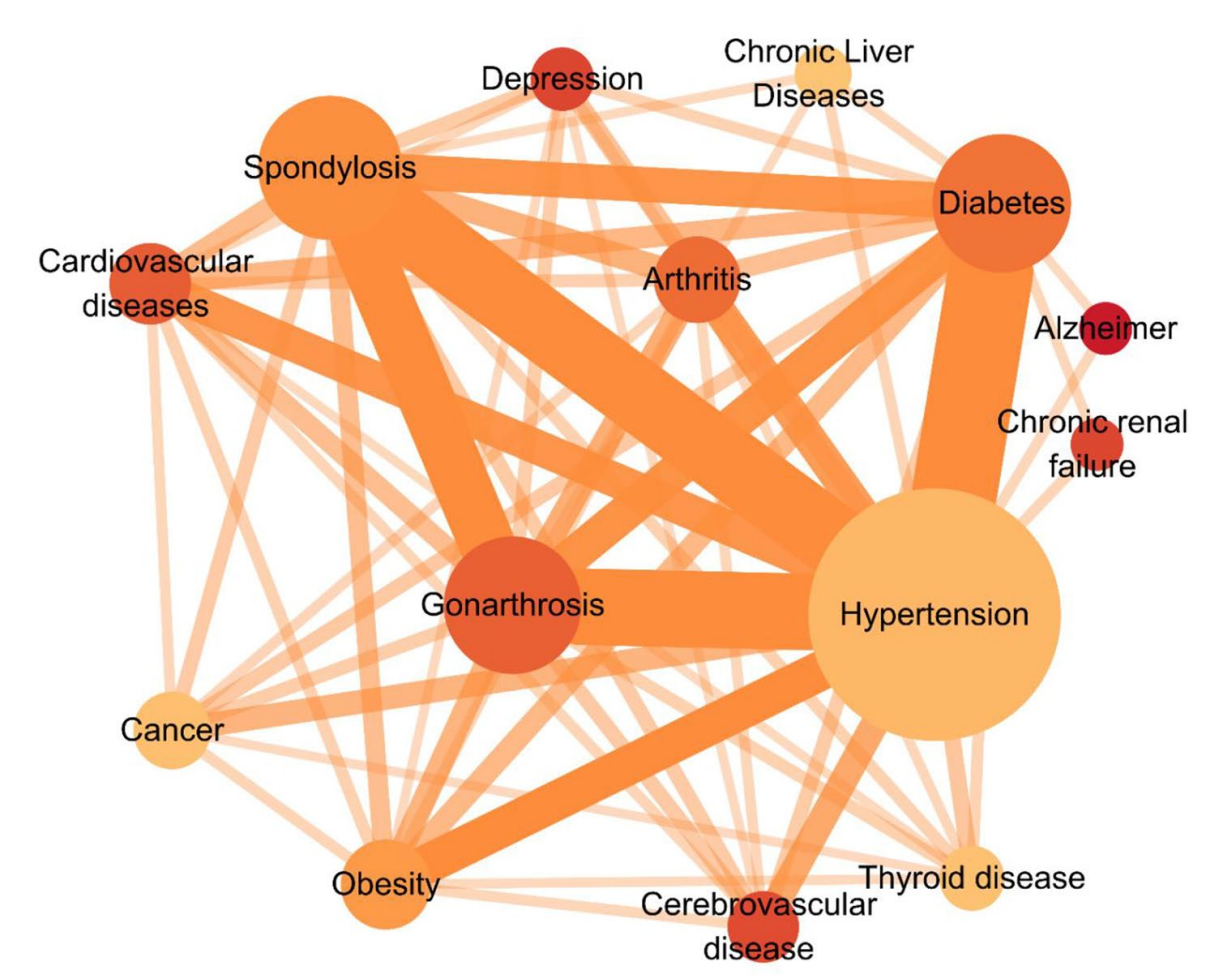
Relation network graph of the overview of the chronic diseases. Note: The minimum number of links that can be displayed is 20. Node size according to the prevalence of the disease, node color according to the percentage of patients with MCD, and the width of the link is based on the number of people with both diseases

The three chronic diseases with the highest prevalence among the 15 surveyed were hypertension (40.2%), spondylosis (18.7%), and diabetes (17.6%). Chronic renal failure (93.9%), depression (90.4%), and cerebrovascular diseases (89.6%) were the top three diseases associated with the largest percentage of MCD among all surveyed chronic diseases. The prevalence and specific relationships for each disease are shown in Fig. 1.

### Association rule analysis of MCD

When conducting association rule analysis, the minimum conditional support was set to 1.5% of the minimum rule, confidence was set to 20%, lift was set to be greater than 1.5, and the maximum number of antecedents was set to 5 items. These parameters were determined after considering the total number of samples and the prevalence of various chronic diseases. Moreover, the two-variable association rules of the same variable are regarded as duplicates, because they always appear together and have no additional predictive power [31], such as (A→ B) and (B→ A). When such duplicates are found, association rules that have a higher confidence are retained.

**Fig. 2:**
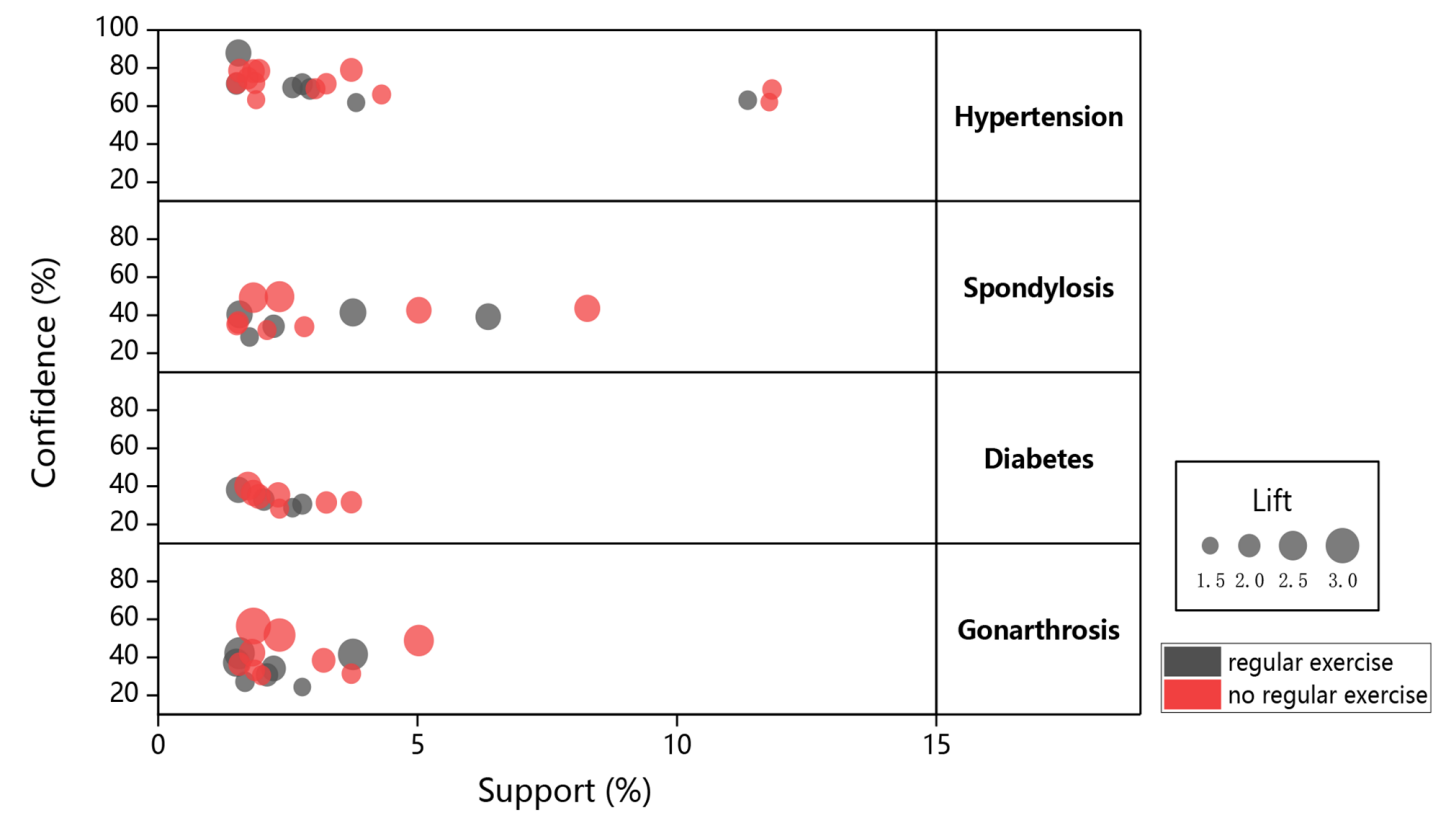
Graph for association rules

In the AG, 23 association rules were identified based on the mining rules we established. In the entirety of the discovered association rules, a total of 4 chronic diseases appeared in the Consequent, whereas 8 chronic diseases appeared in the Antecedent. Figure 2 shows the distribution of all association rules for different consequences. Table 2 describes the specific association rules.

**Table 2:** Significant association rules in AG

Consequent	Antecedent	Confidence (%)	Support (%)	Lift
Diabetes	Cardiovascular Diseases	32.99	2.04	1.83
	Gonarthrosis, Hypertension	30.68	2.78	1.70
	Obesity, Hypertension	38.22	1.55	2.12
	Spondylosis, Hypertension	28.71	2.59	1.59
Hypertension	Cardiovascular Diseases	61.86	3.82	1.56
	Cerebrovascular Disease	69.00	2.93	1.74
	Diabetes	63.13	11.37	1.59
	Diabetes, Spondylosis	69.71	2.59	1.75
	Gonarthrosis, Diabetes	71.58	2.78	1.80
	Obesity, Diabetes	87.95	1.55	2.21
	Obesity, Gonarthrosis	71.72	1.51	1.80
Gonarthrosis	Arthritis	34.20	2.23	2.11
	Cardiovascular Diseases	27.15	1.68	1.67
	Diabetes, Hypertension	24.44	2.78	1.51
	Diabetes, Spondylosis	42.29	1.57	2.61
	Obesity*	30.84	2.10	1.90
	Obesity, Hypertension	37.17	1.51	2.29
	Spondylosis, Hypertension	41.65	3.75	2.57
Spondylosis	Arthritis	34.20	2.23	1.91
	Cardiovascular Diseases	28.52	1.76	1.59
	Gonarthrosis	39.22	6.36	2.19
	Gonarthrosis, Diabetes	40.44	1.57	2.26
	Gonarthrosis, Hypertension	41.45	3.75	2.31

* This association rule appears only in this table

In the IG, 37 association rules were identified based on the mining rules we established. In the entirety of the discovered association rules, a total of 4 chronic diseases appeared in the Consequent, whereas 9 chronic diseases appeared in the Antecedent. Figure 2 shows the distribution of all association rules for different consequences. Table 3 describes the specific association rules.

**Table 3:** Significant association rules in IG

Consequent	Antecedent	Confidence (%)	Support (%)	Lift
Diabetes	Cardiovascular Diseases	35.51	2.31	2.06
	Cardiovascular Diseases, Hypertension*	40.12	1.73	2.33
	Gonarthrosis, Hypertension	31.60	3.72	1.84
	Obesity, Hypertension	34.60	1.94	2.01
	Spondylosis, Hypertension	31.52	3.24	1.83
	Spondylosis, Hypertension, Gonarthrosis*	36.51	1.83	2.12
	Spondylosis, Gonarthrosis	28.30	2.34	1.64
Hypertension	Cardiovascular Diseases	66.12	4.31	1.62
	Cardiovascular Diseases, Diabetes*	74.71	1.73	1.84
	Cardiovascular Diseases, Gonarthrosis*	78.67	1.57	1.93
	Cardiovascular Diseases, Spondylosis*	72.15	1.52	1.77
	Cerebrovascular Disease	69.09	3.03	1.70
	Depression*	63.39	1.89	1.56
	Diabetes	68.78	11.83	1.69
	Diabetes, Gonarthrosis	79.10	3.72	1.94
	Diabetes, Gonarthrosis, Spondylosis*	78.41	1.83	1.93
	Diabetes, Spondylosis	71.76	3.24	1.76
	Gonarthrosis*	62.13	11.78	1.53
	Obesity, Diabetes	78.49	1.94	1.93
	Obesity, Gonarthrosis	72.16	1.86	1.77
Gonarthrosis	Arthritis	38.46	3.19	2.03
	Arthritis, Hypertension*	42.50	1.81	2.24
	Cardiovascular Diseases	30.61	1.99	1.61
	Cardiovascular Diseases, Hypertension*	36.42	1.57	1.92
	Diabetes, Hypertension	31.46	3.72	1.66
	Diabetes, Spondylosis	51.76	2.34	2.73
	Diabetes, Spondylosis, Hypertension*	56.56	1.83	2.98
	Obesity, Hypertension	33.18	1.86	1.75
	Spondylosis, Hypertension	48.84	5.03	2.58
Spondylosis	Arthritis	33.97	2.82	1.72
	Arthritis, Hypertension*	36.25	1.54	1.84
	Cardiovascular Diseases	32.24	2.10	1.64
	Cardiovascular Diseases, Hypertension*	35.19	1.52	1.79
	Diabetes, Hypertension, Gonarthrosis*	49.29	1.83	2.50
	Diabetes, Gonarthrosis	49.72	2.34	2.52
	Gonarthrosis	43.62	8.27	2.21
	Gonarthrosis, Hypertension	42.66	5.03	2.17

* This association rule appears only in this table

There is only one association rule (obesity → gonarthrosis) that was be found in the AG (Table 2). This association rule means that, among middle-aged and older individuals who exercise regularly, 2.1% suffered from obesity and gonarthrosis simultaneously, and the likelihood of obese patients suffering from gonarthrosis was 30.8%, which was 1.91 times that of the average individuals. However, this difference was not significant in the IG.

Fourteen of the 37 association rules discovered in the IG were absent in the AG. It included 2 two-variable association rules: (depression → hypertension) and (gonarthrosis → hypertension), 8 three-variable association rules (cardiovascular diseases, hypertension → diabetes), (cardiovascular diseases, diabetes → hypertension), (cardiovascular diseases, gonarthrosis → hypertension), (cardiovascular diseases, spondylosis → hypertension), (arthritis, hypertension → gonarthrosis), (cardiovascular diseases, hypertension → gonarthrosis), (arthritis, hypertension → spondylosis), (cardiovascular diseases, hypertension → spondylosis), and 4 four-variable association rules (spondylosis, hypertension, gonarthrosis → diabetes), (diabetes, gonarthrosis, spondylosis → hypertension), (diabetes, spondylosis, hypertension → gonarthrosis), (diabetes, hypertension, gonarthrosis → spondylosis).

The frequency of occurrence of all chronic diseases as consequences or antecedents in all the added association rules is shown in Table 4. From Table 4, we can also see that the frequency of cardiovascular diseases, spondylosis, and diabetes increased by 150%, 143%, and 125%, respectively, which are the three chronic diseases with the highest frequency increase. In addition, depression that did not appear before appeared in the AG’s association rules.

**Table 4:** Comparing the differences in association rules between AG and IG

Association rule	Regular exercise			No regular exercise			Increase (%)^a^
	23			37			61
	Consequent	Antecedent	Total	Consequent	Antecedent	Total	
**Spondylosis**	5	2	7	8	9	17	143
**Gonarthrosis**	7	3	10	9	12	21	110
**Hypertension**	7	7	14	13	15	28	100
**Diabetes**	4	4	8	7	11	18	125
**Arthritis**	0	2	2	0	4	4	100
**Cardiovascular diseases**	0	4	4	0	10	10	150
**Cerebrovascular disease**	0	1	1	0	1	1	0
**Obesity**	0	3	3	0	4	4	33
**Depression**	0	0	0	0	1	1	-

a: Percentage improvement in B relative to A. Calculation formula: (B-A)/A. A: Regular exercise group; B: No regular exercise group

## Discussion

Nearly two-thirds of older Germans suffer from MCD according to data on insured people, aged 65 and over, in Germany [32]. Additionally, a survey of the elderly population, aged 60 years and above, in Bangladesh showed that the prevalence of MCD was 53.8%, and the proportion of MCD was higher among women, illiterate individuals, singles, and the poorest population [33]. Data from a total of 660,000 patient visits from 148 primary health care services in the United States found that the prevalence of MCD increased dramatically with age and plateaued at age 80 years, thus exhibiting an S-shape trend. Overall, 45.2% of patients had more than one chronic disease in that study [34]. From our data, the prevalence and disease characteristics of MCD in Korea are consistent with those in other countries. Women, individuals over 65 years old, low education level, no regular exercise habits were associated with significantly higher prevalence of MCD. Importantly, in the face of an ultra-aging population, South Korea must address these issues by 2025 [35]. The individual and economic burdens of MCD are anticipated to rise as South Korea’s population ages; moreover, the aging population is rapidly rising. Developing MCD prevention policies is crucial for reducing these burdens [36].

By examining the relation network graph of the overview of the chronic diseases (Fig. 1), we can observe that metabolic diseases and musculoskeletal disorders primarily occupy the central positions. Certain metabolic diseases directly impact the formation and regenerative capabilities of bone and joint tissues. For instance, metabolic abnormalities associated with elevated blood glucose levels and diabetes can impair the formation and regrowth of skeletal tissues [37]. Moreover, metabolic diseases indirectly influence musculoskeletal health by influencing body weight and inflammatory status. Conversely, musculoskeletal disorders limit patients’ physical activities and mobility, necessitating the long-term use of hormonal medications and non-steroidal anti-inflammatory drugs (NSAIDs). These factors pose significant risks for metabolic diseases [38]. Moreover, it is important to observe in Fig. 1 that both Alzheimer’s disease and depression, although distinct in their types, share a strong correlation with the brain and nervous system. While the overall incidence of these conditions may not be extensive, approximately 90% of individuals diagnosed with Alzheimer’s disease and depression also present MCD. Alzheimer’s disease is specifically linked to metabolic disorders (hypertension and diabetes). Because Alzheimer’s is fundamentally a metabolic disease of the brain that is influenced by insulin and insulin-like growth factor resistance and deficiency, it shares similarities with the effects and consequences of diabetes mellitus [39]. Depression is linked to a wide range of chronic diseases, as depressive disorders not only assume an important role in the development, progression, and outcomes of chronic diseases[40], but also, a healthy psychological state is a significant protective factor against chronic diseases [41].

Based on Table 4, we can observe a substantial increase in the occurrence rate of almost all chronic diseases in the IG compared to the AG. Pervasive chronic conditions share “physical inactivity” as a common risk factor [38] and “systemic low-grade inflammation” as pathogenesis, which may set off a chain reaction leading to the development of a vicious cycle of chronic diseases and poor outcomes. Exercise therapy is distinguished by its anti-inflammatory benefits at the cellular, tissue, and organ levels, as well as its beneficial psychological and physiological effects, including an increase in muscle strength, better blood pressure regulation, and insulin sensitivity [42]. It is recommended as the fundamental form of treatment according to the current clinical guidelines. In the face of MCD, the circumstances become a great deal more complicated. The complexity and heterogeneity of patients with MCD render traditional disease-specific guidelines inadequate and complicate clinical decision-making [43]. The few existing MCD guidelines support a healthy lifestyle, which includes regular physical activities, but does not include specific exercise therapy recommendations [44].

Association rules are popular and powerful data mining techniques that extensively search for hidden patterns, making them suitable for discovering prediction rules involving subsets of the characteristics in a medical data set [45, 46]. When there are several target attributes, illness detection is improved by finding association rules in medical records [47]. The research association rules discovered can provide a theoretical basis and guiding direction for exercise as a treatment plan for MCD.

In the IG, 40.12% of patients with cardiovascular diseases and hypertension also had diabetes, which was 2.33 times the normal value. Similarly, 74.71% of patients with cardiovascular diseases and diabetic also had hypertension, which was 1.83 times the usual number, consistent with the findings of the majority of prior studies that these three diseases are highly linked [46, 48]. The coexistence of hypertension and diabetes has a synergistic effect on cardiovascular damage [49]. Compared with patients with isolated hypertension or diabetes, patients with both conditions have a higher risk of cardiovascular diseases [50], with the incidence of cardiovascular diseases being approximately 4–8 times higher than that of the general population [51, 52]. The relative risk of cardiovascular disease-related mortality increases by 2.5–7.2 times in this population [53]. The coexistence of hypertension and diabetes is not a coincidence, and there may be common underlying causes, but the detailed mechanisms have not been fully elucidated. Insulin resistance may be a key pathogenic factor for the coexistence of diabetes and hypertension [54, 55]. There are studies indicating that insulin resistance is implicated in the pathogenesis and progression of hypertension as well as type 2 diabetes. The low-grade inflammation induced by insulin resistance is considered as the primary mechanism responsible for the development of endothelial dysfunction, hypertension-induced target organ damage, and metabolic abnormalities [56]. In patients with diabetes, the presence of hypertension worsens endothelium-dependent and independent coronary artery blood flow reserve indices [57]. However, these two association rules were not observed in AG, and the connection between them seems to have been “severed.” Given the numerous benefits of exercise [13], it is reasonable to hypothesize that it plays a significant role in “severing” the link between the three. Thankfully, there are enough experimental data exist to test our hypothesis [58, 59]. We are not surprised by the appearance of the (depression → hypertension) association rule in IG. The hyperreactivity of the sympathetic nervous system and genetic influences may be the associated underlying mechanisms [60, 61], as well as the use of antidepressants, which can interfere with blood pressure regulation in patients who are hypertensive by generating blood pressure fluctuations and orthostatic hypotension [62]. However, (depression → hypertension) association rule did not occur in the AG, possibly because exercise reduces self-reported depressive symptoms among patients with hypertension [63]. Of note, (obesity → gonarthrosis) appeared in AG. According to current evidence, people with healthy joints and no joint injuries should be actively encouraged to engage in regular exercise for the benefit of their joints, as well as for their general health [64]. Conversely, if the individual is obese, we must carefully consider the intensity and frequency of the recommended exercise. Excess weight puts additional stress on weight-bearing joints. Each additional kilogram of body mass increases the compressive load on the knee by approximately 4 kg during gait [65]. Thus, patients with obesity must consider safer exercise patterns, including the intensity and frequency, when choosing exercise therapy especially when gonarthrosis is present simultaneously.

With these examples, we have reasons to believe that MCD combinations exclusive to the IG have a greater likelihood of benefiting from exercise therapy and may be better managed with exercise therapy. On the contrary, if the MCD combination appears in AG, such as (obesity → gonarthrosis), then caution must be taken when selecting exercise therapy as a treatment method for these patients. There are two main limitations in this study. Firstly, as a cross-sectional study based on applied data mining, the findings can only reveal correlations among various chronic diseases, rather than causal relationships. Secondly, the variable “habit of regular exercise” in this study is based on self-report by study participants, without clear objective criteria, which may introduce potential biases.

## Conclusions

South Korea must be better prepared for the high prevalence of MCD due to an ultra-aging society by 2025. Not only the medical system, but also related industries, such as insurance, sports, nursing, and welfare, require better preparations. Association rule analysis is effective in studying the relationship between various chronic diseases in patients with MCD. Exercise therapy appears to be a safe and beneficial intervention for improving the physical and psychosocial health of people with MCD. Especially when medical services are restricted or limited, exercise should be widely promoted. The results of this study suggest that exercise therapy that targets patients with MCD may be beneficial.

## Data Availability

The data that support the findings of this study are available from the Korean Health Panel Survey. Restrictions apply to the availability of these data which were used under license for the current study. The data is not publicly available. However, data may be available from the corresponding authors upon reasonable request and with permission from the Korean Health Panel Survey.

## Abbreviations

MCD: Multiple Chronic Diseases
WHO: World Health Organization
KHPS: Korean Health Panel Survey
BMI: Body Mass Index
AG: Active group
IG: Inactive group
